# Stable Adult Hippocampal Neurogenesis in Cannabinoid Receptor CB2 Deficient Mice

**DOI:** 10.3390/ijms20153759

**Published:** 2019-08-01

**Authors:** Leonore Mensching, Nevena Djogo, Christina Keller, Sebastian Rading, Meliha Karsak

**Affiliations:** Center for Molecular Neurobiology (ZMNH), University Medical Center Hamburg-Eppendorf (UKE), 20246 Hamburg, Germany

**Keywords:** G-protein coupled receptor, adult neurogenesis, cannabinoid receptor, CB2, dentate gyrus, subgranular zone, DCX, Ki67, calretinin

## Abstract

The G-protein coupled cannabinoid receptor 2 (CB2) has been implicated in the regulation of adult neurogenesis in the hippocampus. The contribution of CB2 towards basal levels of proliferation and the number of neural progenitors in the subgranular zone (SGZ) of the dentate gyrus, however, remain unclear. We stained hippocampal brain sections of 16- to 17-week-old wildtype and CB2-deficient mice, for neural progenitor and immature neuron markers doublecortin (DCX) and calretinin (CR) and for the proliferation marker Ki67 and quantified the number of positive cells in the SGZ. The quantification revealed that CB2 deficiency neither altered overall cell proliferation nor the size of the DCX+ or DCX and CR double-positive populations in the SGZ compared to control animals. The results indicate that CB2 might not contribute to basal levels of adult neurogenesis in four-month-old healthy mice. CB2 signaling might be more relevant in conditions where adult neurogenesis is dynamically regulated, such as neuroinflammation.

## 1. Introduction

The endocannabinoid system, with its two G protein coupled receptors cannabinoid receptor 1 (CB1) and 2 (CB2), is known to have important functions in the central nervous system. Cannabinoids have been linked to the regulation of adult neurogenesis (AN), a process in the mammalian brain that takes place in stem cell niches in the adult brain and is responsible for the continued generation of new neurons [1]. The largest rates of neurogenesis are seen in the subgranular zone (SGZ) of the dentate gyrus (DG) in the hippocampus and in the subventricular zone. New granule neurons are generated from stem cells located in the SGZ, where they undergo proliferation and maturation and migrate into the granular cell layer (GCL) [2] contributing to learning and memory in the DG [3]. Neural progenitor cells (NPC) are committed to the neuronal fate and express doublecortin (DCX) [4]. DCX+ immature neurons that stopped proliferating additionally express neuronal markers, such as NeuN and calretinin (CR) [4,5]. The DCX+ cell population (type 2b and 3 NPC and immature neurons) is a representative measure for the structural plasticity in the SGZ [6]. In combination with CR, it allows for the determination of the amount of postmitotic immature neurons that may be integrated into the hippocampal network [7].

The involvement of the endocannabinoid system in AN processes such as proliferation, differentiation, survival, and migration of NPCs has been reported [8,9]. CB2 is expressed on NPCs in vitro and in vivo and stimulation of NPCs with CB2 agonist HU308 leads to increased proliferation and neurosphere formation by the engagement of PI3K/Akt/mTORC1 and ERK1/2 pathways [8,10,11,12]. It has been reported that CB1–CB2 heterodimers contribute to the proliferative effect of endocannabinoids in the SGZ [13]. The treatment with HU308 resulted in an increase in cell proliferation in the SGZ in wildtype mice (measured by BrdU staining) that is absent in CB2 knockout animals and an excitotoxicity-induced increase in neurogenesis via kainic acid was also reduced in CB2-deficient animals [11,12]. Palazuelos et al. (2006) [11] also showed reduced basal levels of cell proliferation in the SGZ in CB2 knockout mice at embryonic day 17.5 and eight weeks postnatally. Upon treatment with the CB2 antagonist/inverse agonist SR144528, wildtype animals displayed reduced SGZ cell proliferation, which was comparable to that of CB2-deficient mice [11], indicating an involvement of basal CB2 signaling in the regulation of proliferation rates in the SGZ. Furthermore, in a subsequent study of the same group the importance of mTORC1 signaling in the CB2-induced an increase in NPC proliferation in vitro and in eight-week-old mice was demonstrated [12]. 

In two different disease models, CB2 has also been shown to impact neurogenesis positively. In a mouse model for HIV-1 infection where mice exhibit decreased AN in the hippocampus, treatment with a CB2 agonist rescues the impaired phenotype [14]. After a cortical stroke the activation of CB2 promotes neuroblast migration to and differentiation at the infarct site [15].

Cannabinoids, as regulators for AN, are an emerging topic in neurogenesis research. The role of CB2 in NPC proliferation could act in synergy with other neuroprotective effects associated with CB2 in the context of neurodegeneration, where impairment of AN is a common hallmark [8,9,16,17].

Although the proliferative effect of CB2 stimulation on NPC in the SGZ has thus been repeatedly demonstrated [11,12,13], the question about the possible contribution of CB2 to basal SGZ proliferation and the size of NPC populations has not been directly addressed. Accordingly, the aim was to decipher the influence of CB2 presence on the size of the DCX+ NPC population, the abundance of postmitotic immature neurons (DCX+/CR+) and the overall cell proliferation (Ki67+) in the SGZ of the DG at a basal and stable state in healthy adult mice. It was shown here, that in CB2-deficient four-month-old animals neither the size of the investigated NPC populations nor overall cell proliferation in the SGZ of the dentate gyrus was altered compared to wildtype animals.

## 2. Results

**Figure 1:**
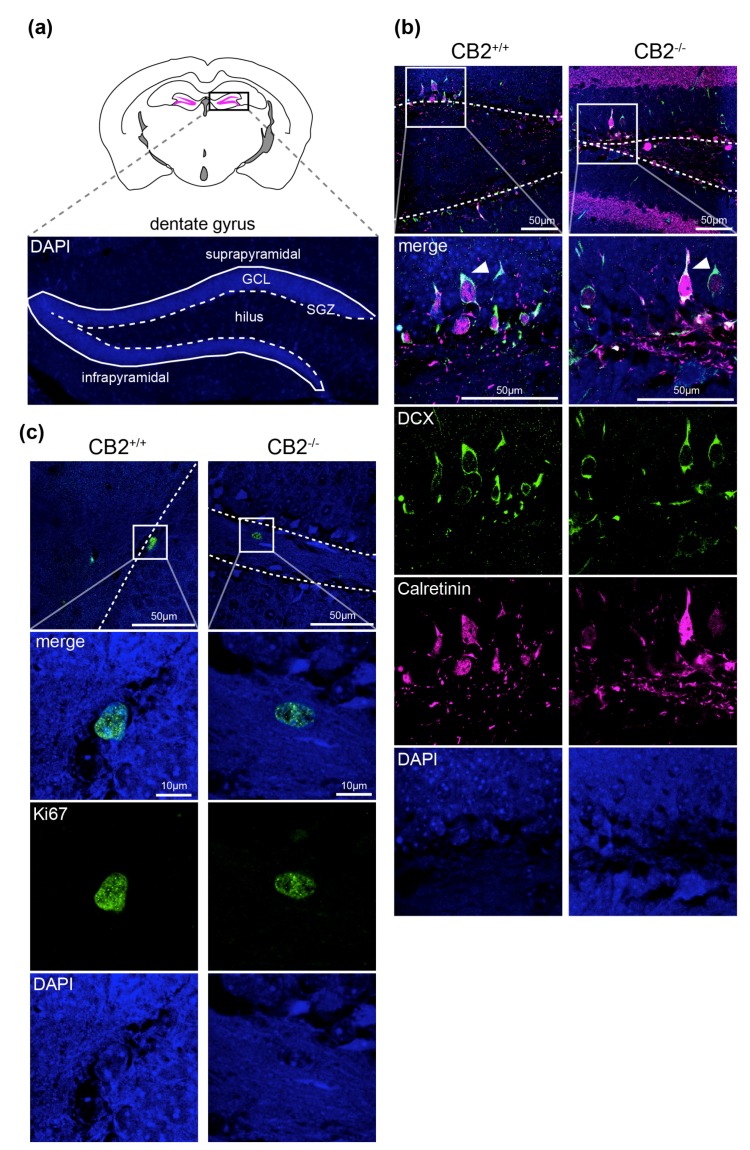
Representative confocal microscopy images of immunohistochemistry detection of Ki67, DCX (doublecortin) and CR (calretinin) in CB2 wildtype and knockout brain sections. (**a**) Overview of the mouse dentate gyrus (magenta in black rectangle) in the hippocampus and identification of relevant structures in a representative microscopy image of a brain section with DAPI-staining. (**b**) Merged fluorescent images of subgranular zone (SGZ) from both genotypes stained with DCX (green), calretinin (magenta) and DAPI (blue) are seen in the first row. The white rectangle shows the field of view that is shown in rows two to four in higher magnification. White arrowheads indicating the location of CR+DCX+ cells, the asterisk shows a CR+ inhibitory neuron in the hilus. (**c**) Merged fluorescent images of SGZ from both genotypes stained with Ki67 (green) and DAPI (blue) are seen in the first row. The white rectangle shows the field of view that is shown in rows two to three in higher magnification. The dotted line represents the SGZ. Template for scheme in (**a**) modified from The Scalable Brain Atlas [18,19].

For the quantification of intermediate neuronal progenitors (DCX+CR−) and postmitotic immature neurons (DCX+CR+) 40 μm brain sections were co-stained for the neuronal progenitor markers DCX and CR, which is expressed by immature neurons together with DCX (Figure 1). Immunostaining of brain sections with the proliferation marker Ki67 was used to quantify the number of dividing cells.

Positive cells were counted in the SGZ of the DG and normalized to the sample volumes in mm^3^ of the infra- (ventral) and suprapyramidal (dorsal) blades of the GCL and SGZ (Figure 1a). Figure 1 shows representative stainings for DCX/CR (Figure 1b) and Ki67 (Figure 1c) from brain sections of CB2-deficient mice and wildtype littermates. In both genotypes the cellular distribution of the fluorescent signals were identical. Ki67 was detected in the nucleus (Figure 1c), whereas DCX signal was seen in the cytosol (Figure 1b). Positive cells were distributed along the full length of the SGZ and in both DG blades. In DCX and CR double positive cells (Figure 1c, white arrows), CR staining was predominantly seen in the nucleus. CR positive inhibitory neurons were significantly larger than neuronal progenitors and located mainly in the deep hilus (asterisk, Figure 1b).

**Figure 2:**
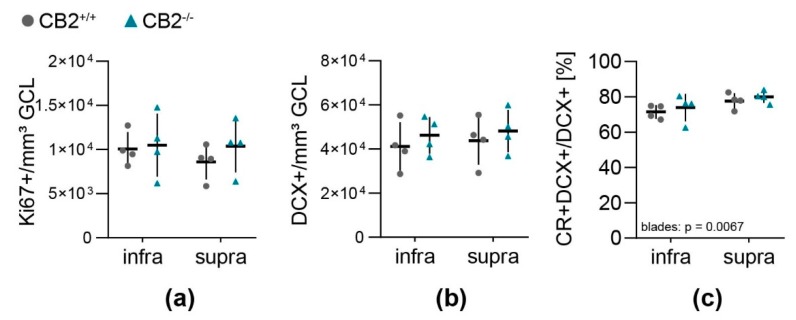
Quantification of neural progenitor cell populations and proliferation in the dentate gyrus of wildtype and CB2-deficient mice. The number of cells positive for Ki67 (**a**) and DCX (**b**) per sample volume (GCL and SGZ) in the infra- and suprapyramidal blade of the dentate gyrus in CB2^+/+^ (circle) and CB2^−/−^ (triangle). (**c**) Percentage of post-mitotic DCX+/CR+ immature neurons from total amount of DCX+ cells in the infra- and suprapyramidal blade of the dentate gyrus in both genotypes. Each *n* represents one animal. CB2^+/+^, *n* = 4, CB2^−/−^, *n* = 4. Mean ± SD.

Morphological investigation of the brain sections showed no difference in the distribution or location of DCX, CR or Ki67 positive cells in the DG between the genotypes. These observations corroborate the results of the cell population quantification (Figure 2). 

Overall cell proliferation per GCL and SGZ volume (Figure 2a) did not differ between the blades of the DG or between genotypes. Neither did the amount of DCX+ type 2b and 3 NPC (Figure 2b), that was four times larger than the number of Ki67+ proliferating cells per GCL-SGZ volume (Table 1). Around three quarters of all DCX positive cells were postmitotic immature neurons (DCX+CR+) (Figure 2c, DCX+CR+/DCX+). In the infrapyramidal blade there was an overall lower percentage of double positive cells compared to the suprapyramidal blade of the DG (Table 1, *p* = 0.0067).

## 3. Discussion

The quantification of adult hippocampal neurogenesis in the SGZ of CB2-deficient and wildtype mice in this work revealed that CB2-deficiency did not seem to influence the size of the DCX+ neural progenitor cell population, the relative frequency of postmitotic immature neurons (DCX+/CR+) or the overall cell proliferation. With the applied quantification method data were gathered which are in agreement with previously published reports, stating that in the mouse SGZ there are around four times more DCX+ than Ki67+ cells [20] and that the proportion of DCX+/CR+ immature neurons represents around 70% to 80% of all DCX+ cells [21].

Previous research consistently showed the effect of CB2 agonist treatment on NPC proliferation in the SGZ of adult mice. On the contrary, the question about a possible contribution of basal CB2 signaling for adult neurogenesis remains somehow unclear [11,12]. In fact, two studies of the same group reported in part inconsistent data on this issue. In the first study, a reduction of about 30% of BrdU positive cells was demonstrated in the SGZ of eight-week-old CB2-deficient mice [11]. In the second study [12], which, in principle, focused on the signal transduction mechanism involved in CB2 receptor induced neurogenesis, again basal SGZ cell proliferation data in eight-week-old vehicle-treated mice of the *Cnr2^tm1Zim^* strain were included, which, however, showed no obvious BrdU differences between vehicle-treated wildtype and CB2 knockout mice in an excitotoxicity-model. In line with these later observations of this group, we have now confirmed that CB2 deficiency did not influence measured adult neurogenesis parameters in *Cnr2^tm1Dgen^* transgenic mice at the age of four months. The most important differences between this study and previous neurogenesis research in CB2 knockout mice [11,12] are the ages of the animals, the transgenic mice that were used and the labeling of proliferating cells with Ki67 instead of the synthetic thymidine analogue BrdU. We investigated four-month-old mice as we wanted to investigate the contribution of basal CB2 signaling to processes in stable adult neurogenesis and, until the age of two to three months, cell proliferation rates in the mouse SGZ are still highly dynamic [20]. Avoiding the use of BrdU, which requires repeated handling of the animals when injected, the staining of Ki67 excludes possible unspecific side effects in neural stem cells due to substance treatment and handling stress [22].

In this study, 16- to 17-week-old animals from the *Cnr2^tm1Dgen^* transgenic mouse strain were investigated instead of eight-week-old animals from the *Cnr2^tm1Zim^* strain [11,12,21]. *Cnr2^tm1Dgen^* have a deletion in the N-terminal region of the gene coding for CB2, whereas *Cnr2^tm1Zim^* have an insertion of a neomycin cassette into the C-terminal region of the gene. It is possible that *Cnr2^tm1Zim^* animals express inactive truncated CB2 protein interfering with signaling from other receptors [23]. 

Given that the interaction of CB1 and CB2 is relevant for the proliferative effect of endocannabinoids in the SGZ [13], it is of concern that the use of *Cnr2^tm1Zim^* knockout animals might have led to unspecific effects, possibly influencing proliferation rates in the SGZ in these mice [11].

Although no effect of basal CB2-mediated signaling on adult neurogenesis was seen in this study, it cannot be concluded that CB2 is irrelevant for the regulation of AN. The afore-mentioned interaction of CB1 and CB2 in promoting neurogenesis [13] might be of high importance here. To a certain degree, CB1 signaling might compensate for the lack of CB2, so basal levels of neurogenesis might not have been affected. CB1-deficiency alone has been implicated in reduced baseline neurogenesis [24,25] and could be a more relevant factor in basal adult neurogenesis than CB2. 

Additionally, in former publications CB2 activation has been shown to promote NPC proliferation in the DG in an inflammatory model of viral infection [14], and blocking CB2 decreases the migration and differentiation of NPCs at the injury site in a murine stroke model [15]. The role of CB2 in neurogenesis might be more important in neuroinflammatory conditions and in response to pathophysiological stimuli than in maintaining basal AN levels. 

It remains to be determined if and how CB2 expression on neural progenitors is regulated to influence neurogenesis and if CB2-mediated effects on other cells, such as microglia, might play a role in the observed effects of CB2 stimulation in in vivo studies [15,26,27].

## 4. Materials and Methods 

### 4.1. Animals

All mice used in this study were housed in a 12 h:12 h light-dark cycle with water and food pellets ad libitum at a temperature of 21 °C. All the mice were derived from heterozygous breeding pairs and homozygous CB2 knockouts and wildtype littermates were used for the studies. Housing facilities and animal handling were in accordance with the German and European Community laws on protection of experimental animals and approved by the Behörde für Gesundheit und Verbraucherschutz of the City of Hamburg (project identification code number 54/16; date of approval 14 July 2016).

### 4.2. Preparation of Mouse Brain Sections

Eight female 16- to 17-week-old *Cnr2^tm1Dgen^* CB2-deficient and wildtype mice were deeply anesthetized with 120 μg/g body weight esketamine and 16 μg/g body weight xylazine intra-peritoneally in sterile physiological 0.9% NaCl solution and then transcardially perfused with 25 mL 4% PFA. Whole brains were harvested and stored in 4% PFA at 4 °C until use, but at least 24 h. Fixed brains were cut on a vibratome at 40 μm section thickness from the rostral beginning of the hippocampus until the caudal end in a series of ten. Sections were collected in 24-well plates filled with cryoprotectant solution (50% *v*/*v* 0.1 M phosphate buffer (26.5 mM NaH_2_PO_4_, 77 mM Na_2_HPO_4_, 300 mM NaCl in ddH_2_O, pH 7.2), 30% *w*/*v* sucrose, 1% *w*/*v* polyvinylpyrrolidone, 30% *v*/*v* ethylene glycol in ddH_2_O) at 4 °C until immunohistochemistry.

### 4.3. Immunohistochemistry of Mouse Brain Sections

One series of 10 brain sections (distance between sections: 400 μm) per animal were transferred from cryoprotectant to cold PBS in 24-well plates and washed three times. Antigen retrieval was performed in 10 mM sodium citrate (in ddH_2_O, pH 9) at 80 °C in an incubator for 30 min. Tissue sections were then cooled at room temperature, washed with PBS and blocked with IHC blocking solution (5% normal goat serum, 0.2% Triton^®^ X-100, 0.02% sodium azide in PBS) for 45 min at room temperature on a shaker, and then incubated with primary antibody against DCX (rabbit anti-DCX, Synaptic Systems, Göttingen, Germany #326003, 1:700) and CR (guinea pig anti-CR, Synaptic Systems, Göttingen, Germany, #214104, 1:500) or against Ki67 (rabbit anti-Ki67, Thermo Fisher Scientific, Schwerte, Germany, #PA5-19462, 1:500) in PBS overnight at 4 °C on a shaker. After incubation with the primary antibody, brain sections were washed three times with PBS each for 5 min on a shaker at room temperature. Fluorescent-dye conjugated secondary antibodies (anti-rabbit IgG Alexa Fluor^®^ 488, anti-guinea pig IgG Alexa Fluor^®^ 594, Thermo Fisher Scientific, Schwerte, Germany, #R37116 and #A-11076, 1:500) were prepared in PBS according to used primary antibodies and sections were incubated with the secondary antibody solution for 2 h in the dark at room temperature on a shaker. Sections were again washed three times with PBS each for 15 min on a shaker and then carefully transferred to objectives. ProLong™ Gold Antifade Mountant with DAPI (Thermo Fisher Scientific, Schwerte, Germany, #P36935) was applied and cover slips were carefully placed on the sections. The mounted sections were left to dry at room temperature in the dark for about 24 h and then transferred to boxes and stored at 4 °C until imaging.

### 4.4. Quantification of Neuronal Progenitor Cell Populations and Cell Proliferation

Blinded quantification of DCX+, DCX+/CR+ and Ki67+ neuronal progenitor cells in the subgranular zone of the dentate gyrus in 40 μm-thick brain sections was carried out on an epi-fluorescence microscope using the software tool Stereo Investigator (MBF Bioscience, Williston, VT, USA). For each section and brain hemisphere an overview image of the complete DG with clearly distinguishable infra- and suprapyramidal blades was acquired using the DAPI staining to determine if the hemisphere is quantifiable. Exclusion criteria were the overlapping or damage of tissue. In each hemisphere and blade all positive (DCX+, Ki67+) and double-positive cells (DCX+/CR+) in the SGZ were counted in a focal plane of 20 μm. Using ImageJ [28], the area of the complete GCL and SGZ of the DG was calculated for each hemisphere and blade separately. The total sample volume was then determined by multiplying the GCL area with the thickness of the focal plane (20 μm). All hippocampal sample volumes and cell counts were summed for each animal and positive counts per volume (in mm^3^) for DCX+ and Ki67+ were determined. From DCX+/CR+ counts, the percentage of double-positives from all DCX+ cells were calculated for each animal.

### 4.5. Statistical Analysis

All statistical analysis was carried out using Prism 8.0.1 (GraphPad Software, San Diego, CA, USA) and applied statistical test to estimate differences between sample groups. Values from infra- and suprapyramidal blades are paired and were analyzed using a two-way ANOVA with repeated measures and Sidak-adjusted *p*-values.

## Figures and Tables

**Table 1:** Mean ± SD values of Ki67+ and DCX+ cells per mm^3^ of the granular cell layer and percentage of immature neurons (CR+DCX+) from all DCX+ positive cells in wildtype (CB2^+/+^) and CB2-deficient (CB2^−/−^) mice and in both blades of the dentate gyrus. inf.—infrapyramidal, sup.—suprapyramidal.

Genotype	Ki67+ per mm^3^			DCX+ per mm^3^			CR+DCX+/DCX+ [%]		
	inf.	sup.	both	inf.	sup.	both	inf.	sup.	both
CB2^+/+^	10,060 ± 1921	8604 ± 1971	9174 ± 1737	41,188 ± 10,908	46,220 ± 8353	42,739 ± 10,794	71.49 ± 3.86	77.68 ± 4.4	75.25 ± 3.69
CB2^−/−^	10,504 ± 3564	10,388 ± 2972	10,443 ± 3192	43,837 ± 10,917	48,191 ± 9600	47,390 ± 9028	73.92 ± 7.8	80.00 ± 3.38	77.63 ± 5

## Abbreviations

AN	Adult neurogenesis
CB1	Cannabinoid receptor 1
CB2	Cannabinoid receptor 2
CR	Calretinin
DCX	Doublecortin
DG	Dentate gyrus
GCL	Granular cell layer
SD	Standard deviation
SGZ	Subgranular zone
NPC	Neural progenitor cells
